# REFOLDdb: a new and sustainable gateway to experimental protocols for protein refolding

**DOI:** 10.1186/s12900-017-0074-z

**Published:** 2017-04-24

**Authors:** Hisashi Mizutani, Hideaki Sugawara, Ashley M. Buckle, Takeshi Sangawa, Ken-ichi Miyazono, Jun Ohtsuka, Koji Nagata, Tomoki Shojima, Shohei Nosaki, Yuqun Xu, Delong Wang, Xiao Hu, Masaru Tanokura, Kei Yura

**Affiliations:** 1 Center for Information Biology, National Institute of Genetics, 1111 Yata Mishima, Shizuoka, 411-8540 Japan; 2 The Department of Biochemistry and Molecular Biology, Biomedicine Discovery Institute, Monash University, Clayton, VIC 3800 Australia; 3 Laboratory of Protein Synthesis and Expression, Institute for Protein Research, Osaka University, Suita, Osaka 565-0871 Japan; 4 Laboratory of Structural Biology and Food Biotechnology, Department of Applied Biological Chemistry, Graduate School of Agricultural and Life Sciences, The University of Tokyo, 1-1-1 Yayoi, Bunkyo-ku, Tokyo, 113-8657 Japan; 5 Graduate School of Humanities and Sciences, Ochanomizu University, 2-1-1 Otsuka, Bunkyo, Tokyo, 112-8610 Japan; 6 Center for Simulation Science and Informational Biology, Ochanomizu University, 2-1-1 Otsuka, Bunkyo, Tokyo, 112-8610 Japan; 7 School of Advanced Science and Engineering, Waseda University, 3-4-1 Okubo, Shinjyuku, Tokyo, 169-8555 Japan

**Keywords:** Solubilization, Inclusion body, Refolding, Renaturation, Crystallization

## Abstract

**Background:**

More than 7000 papers related to “protein refolding” have been published to date, with approximately 300 reports each year during the last decade. Whilst some of these papers provide experimental protocols for protein refolding, a survey in the structural life science communities showed a necessity for a comprehensive database for refolding techniques. We therefore have developed a new resource – “REFOLDdb” that collects refolding techniques into a single, searchable repository to help researchers develop refolding protocols for proteins of interest.

**Results:**

We based our resource on the existing REFOLD database, which has not been updated since 2009. We redesigned the data format to be more concise, allowing consistent representations among data entries compared with the original REFOLD database. The remodeled data architecture enhances the search efficiency and improves the sustainability of the database. After an exhaustive literature search we added experimental refolding protocols from reports published 2009 to early 2017. In addition to this new data, we fully converted and integrated existing REFOLD data into our new resource. REFOLDdb contains 1877 entries as of March 17^th^, 2017, and is freely available at http://p4d-info.nig.ac.jp/refolddb/.

**Conclusion:**

REFOLDdb is a unique database for the life sciences research community, providing annotated information for designing new refolding protocols and customizing existing methodologies. We envisage that this resource will find wide utility across broad disciplines that rely on the production of pure, active, recombinant proteins. Furthermore, the database also provides a useful overview of the recent trends and statistics in refolding technology development.

## Background

Establishment of heterologous expression technology of recombinant proteins has revolutionized protein purification such that it is performed with cloned, recombinant proteins expressed in a suitable host. The predominant host is *Escherichia coli*. However, many overexpressed proteins in *E. coli* are found in an insoluble form called inclusion bodies (IBs). Since the target protein is often highly pure in washed IBs, the challenge is not so much to purify the target, but rather to solubilize IBs and refold the protein into its native, biologically active state [1, 2]. While many of the operations to prepare IBs are quite general—expression, cell disruption, IB isolation and washing, the precise conditions that are required to achieve efficient refolding vary for each protein.

The refolding experiments consist of two steps: (1) the solubilization of IBs by adding a denaturant and (2) the renaturation of the denatured protein by lowering the denaturant concentration. The solubilization step is relatively easily, performed by adding a denaturant, typically urea or guanidinium chloride at a final concentration of 6–8 M or 6 M, respectively. The renaturation step is often difficult. In order to maximize the refolding yield, the optimization of the following experimental methods/conditions of this process is required:

- refolding method: dilution and dialysis [3], gel filtration column chromatography, column adsorption and desorption [4], and high pressure [5] represent the most common methods. These methods are used to lower the denaturant concentration and allow protein refolding in an aqueous buffer.
- pH: In general, pI should not be used for refolding experiment to avoid isoelectric point precipitation [6].
- temperature: Temperature has an effect on the stability and mobility of the refolding intermediates [7].
- protein concentration: Protein concentration determines the degree of “crowding” and thus the frequency of molecular collisions between the unfolded molecules as well as folding intermediates, which can promote aggregation [8].
- additive(s): Some compounds may stabilize the refolding intermediates and avoid aggregation [9].

Because the suitable refolding methods/conditions differ from protein to protein, a knowledge database of optimized refolding methods/conditions for each protein is an important resource for many biochemists and molecular biologists. Thus, the REFOLD database established and published by Monash University in 2006 played an important role as the sole information source for refolding experiments [10–13]. This database, however, suspended its updates in 2009. We carried out a preliminary study in 2013 for the development of a sustainable database on protein refolding technologies. We decided that a new database, REFOLDdb, was required as a gateway to experimental methodologies that describe experimental refolding in detail. We therefore designed a simple data format and consistent data representation among entries so that users are able to easily interrogate the database and painlessly retrieve and understand search results. The design also allows straightforward maintenance, allowing the database to be sustainable over a long period.

The sustainability of biological databases is a serious issue [14, 15] and database developers have to analyze cost-effectiveness in advance. In the case of databases relating to technologies (Tech_db), the data volume will not expand as rapidly as in the case of molecular databases, for example the International Sequence Database [16], the Worldwide Protein Data Bank [17], UniProt [18], SUPERFAMILY database [19]. Nevertheless, developer of Tech_db must be sensitive to the direct and indirect cost of data extraction from the primary articles, curation and updating. REFOLDdb is designed to balance both cost and usefulness.

We have captured the refolding data from up-to-date literature as well as retrospectively from articles published since 2009. We also updated, converted and integrated the data stored in the REFOLD database into REFOLDdb. As of March 17^th^, 2017, REFOLDdb provides users with data on 1877 experimental methods for refolding 1628 proteins. Most of these data were extracted from 1232 publications.

### Construction and content

We searched the NCBI PubMed database by a keyword search of “(refolding[All Fields] OR renaturation[All Fields]) AND (“proteins”[MeSH Terms] OR “proteins”[All Fields] OR “protein”[All Fields])” to find 2606 research reports published between 2009-early 2017 that might be relevant to REFOLDdb. Manual inspection of the results identified 420 reports that contained experimental protocols for the refolding of 650 proteins. These data were then integrated in REFOLDdb along with the data stored in the REFOLD database. REFOLDdb refers to 1232 publications in total (Full list available via a menu “List of publications referred by REFOLDdb” in “About” page at http://p4d-info.nig.ac.jp/refolddb/about.cgi?lang=EN).

Due to the standardization and other extension of the data format, the database now contains the following functionality: (1) it is searchable by sequence similarity; (2) it is equipped with statistics that enables the discovery of trends in refolding techniques; and (3) it is easy to upload/submit new data to the database manager. Specifically, the database has the following three sections: Article [title/abstract/PubMed ID/Author/Journal/Date], Protein [Protein name/Amino acid sequence/Comment/UniProt ID/Function/Domain] and Experiment [Refolding methods/pH/Temperature/Validation]. We did not itemize “protein concentration” and “additive(s)”, because “protein concentration” is often missing in articles and the description of “additive(s)” is quite heterogeneous. REFOLDdb is composed of 12 tables in a relational database system.

REFOLDdb was created using open-source PostgreSQL relational database server software version 9.2.14 (https://www.postgresql.org/), running under CentOS 7 Server (version 7.2-1511) on a virtual machine based on VMware ESXi (http://www.vmware.com/products/esxi-and-esx.html). The system complies with the security policy of the National Institute of Genetics, Japan. A web-based query interface to the database was developed using the Perl programming language and PDO database abstraction classes (http://jp2.php.net/manual/en/book.pdo.php), and is hosted on the same virtual machine running the Apache 2.4.6 web server.

## Utility and discussion

The top page of REFOLDdb is composed of (a) a horizontal bar menu and (b) a large main search window.

**Fig. 1:**
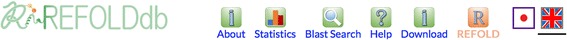
The horizontal menu bar of REFOLDdb

- The horizontal bar menu
  The menu includes 7 icons, “REFOLDdb”, “About”, “Statistics”, “Blast Search”, “Help”, “Download” and “REFOLD”, corresponding to options of database operations and 2 icons for language selection as shown in Fig. 1.

**Fig. 2:**
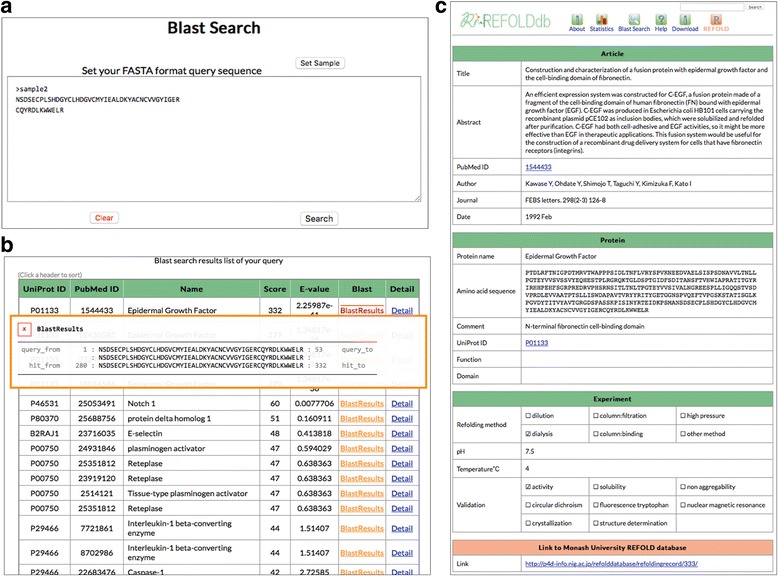
Results of “Blast Search” by one of the three sample amino acids sequences (the middle length). **a**) The blast search window with a sample AAseq. **b**) The result in a table format and the alignment of AAseqs of the query and the top hit in the table. **c**) Details of the top hit
  “HELP”, “DOWNLOAD”, and “REFOLD” allow: browsing a compact manual for the utilization of REFOLDdb featuring screen captures, downloading the data contents of REFOLDdb in a tab-separated values (TSV) file, and accessing the previous REFOLD database respectively.
  A “Statistics” page provides the user with an overview on REFOLDdb records and also a search interface. The pie and bar charts are clickable to retrieve the relevant data entries from REFOLDdb. The histogram of refolding methods developed/used is exemplified in Fig. 3a. It is obvious in the bar chart that “dilution” is the most popular methods and “high_pressure” is rarely used. It is straightforward to become familiar with “high_pressure” method by clicking the bar in Fig. 3a. The data entries that contribute to the bar are displayed in a sortable table. The top 5 ~ 65 records in the table are shown in Fig. 3b. The user is able to directly reach the full description of the method in the database using the “Detail” button in the table and then the original articles, e.g. “A class-A GPCR solubilized under high hydrostatic pressure retains its ligand binding ability” [20].

  “REFOLDdb” at the left end of the menu is a back button for the REFOLDdb user to return to the top page after several operations. “About” refers to a brief introduction of REFOLDdb and the REFOLD database. “Statistics” introduces the anatomy of REFOLDdb by graphically displaying the numbers of experiments by journals, refolding methods developed/used, pH, temperature, protein size, methods for validation, and also the number of refolding experiments by year. In addition, a “Statistics” page provides a search function, which is explained below.
  “Blast Search” page accepts a an amino acid sequence (AAseq) in FASTA format to search for “similar” proteins that were successfully refolded. A sub-menu “set sample” placed just above the blast search window toggles short, medium and long AAseqs for a quick trial (Fig. 2a). Figure 2b introduces the blast search result in a table format by choosing the medium length AAseq in the sample set. By clicking “Blast Results” in the table, the alignment between the query sequence and AAseqs in the database is displayed as shown by the box that overlaps the table. “Detail” button navigates the user to the full record as shown in Fig. 2c. The full record includes a link to the corresponding record in the REFOLD database, if available.

**Fig. 3:**
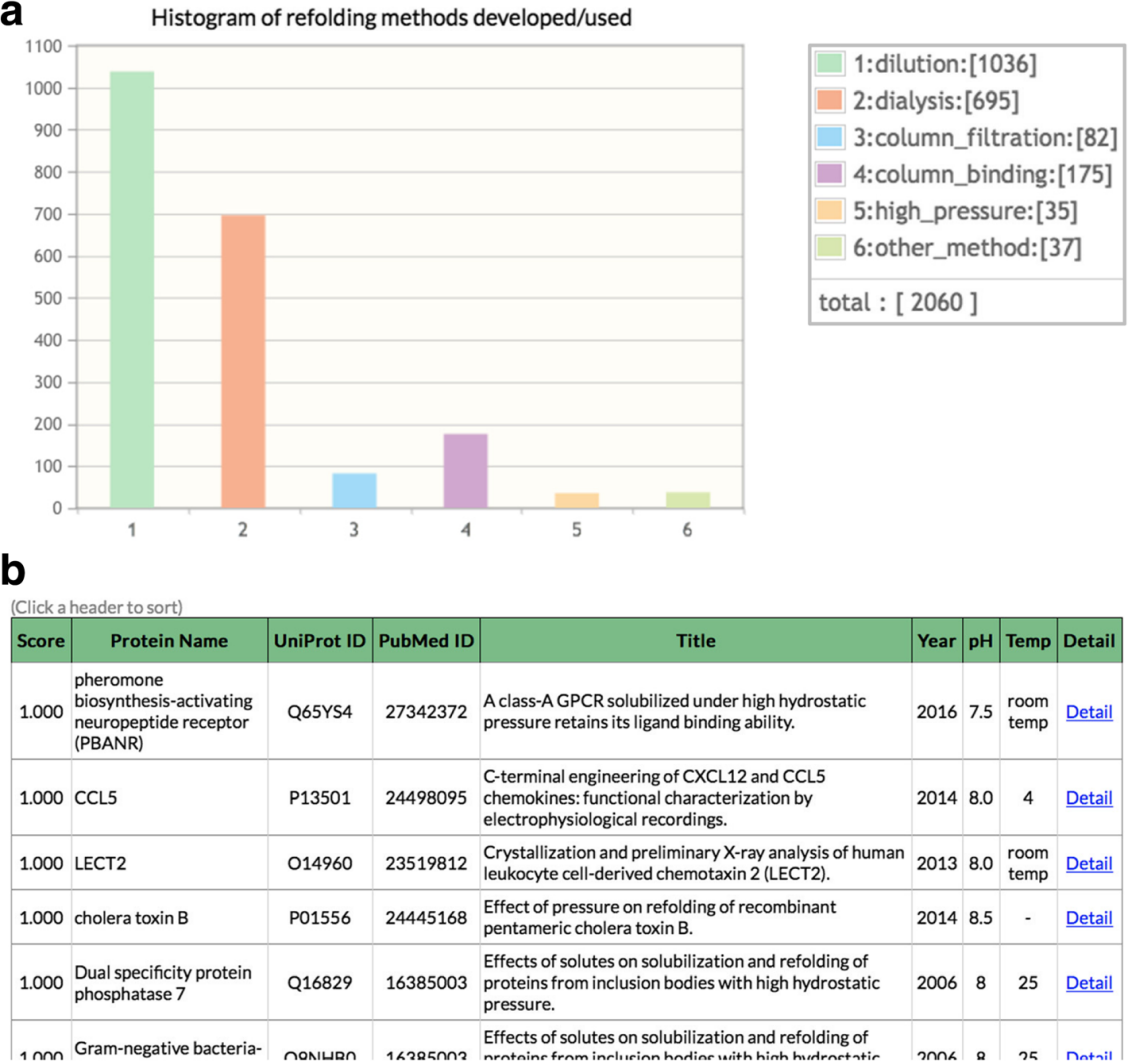
Statistics for data retrieval. **a**) Histogram of refolding methods developed/used. **b**) A part of 35 REFOLDdb entries that compose the “high-pressure” bar in the Fig. 3a
  “Statistics” on experimental conditions such as pH (Fig. 4) and temperature (Fig. 5) might be useful for protein crystallographers: the histogram in Fig. 4 suggests that protein refolding experiments are most successful in a pH range of 7 to 10 regardless of other factors; the histogram in Fig. 5 shows that protein refolding experiments have been mainly performed at two temperature ranges of 0.0–4.9° Celsius (~55%) and 25.0–29.9° Celsius (~23%). We envisage that the database may allow the identification of certain refolding conditions, such as low or high temperature, which may aid downstream crystallization attempts.

**Fig. 4:**
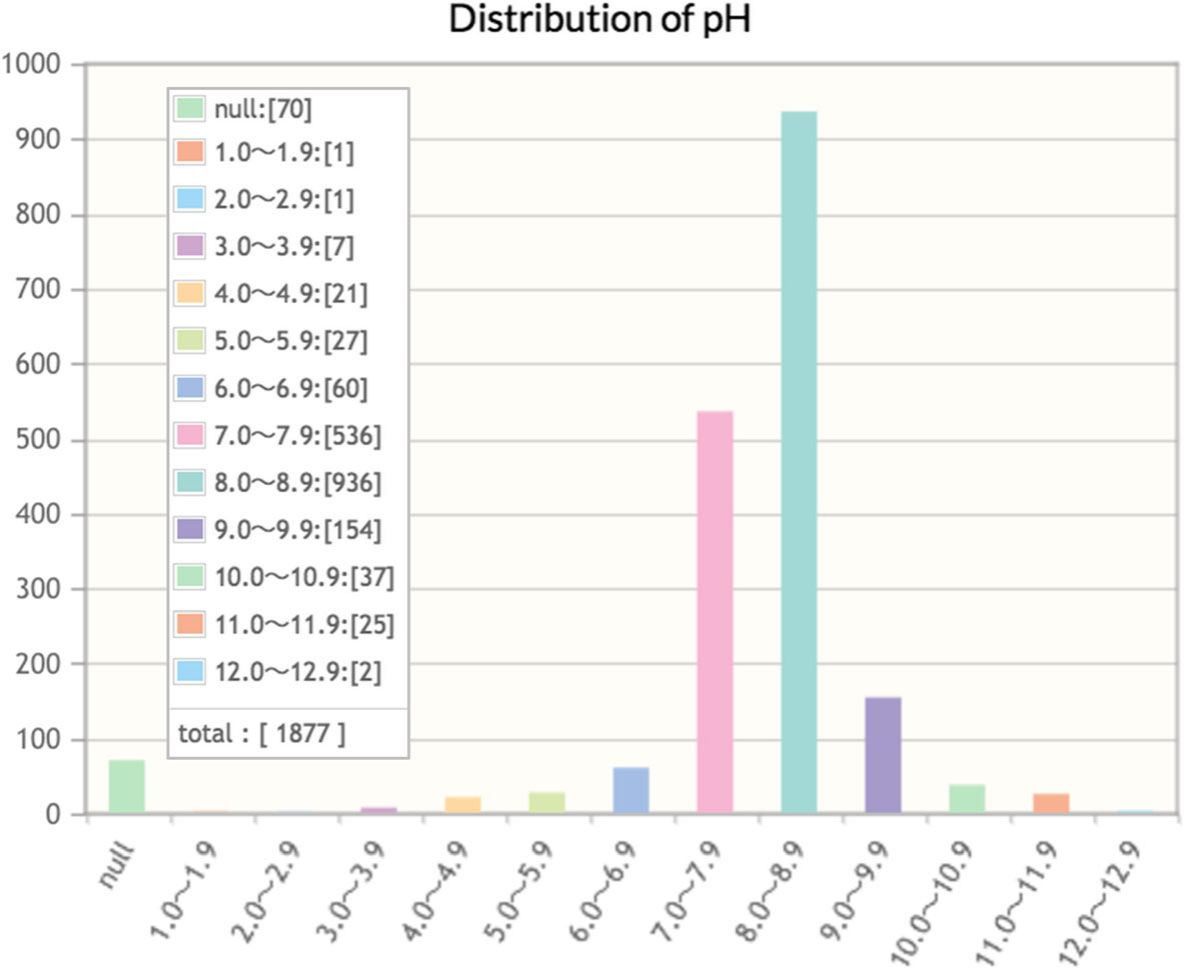
Statistics on an experimental condition, pH

**Fig. 5:**
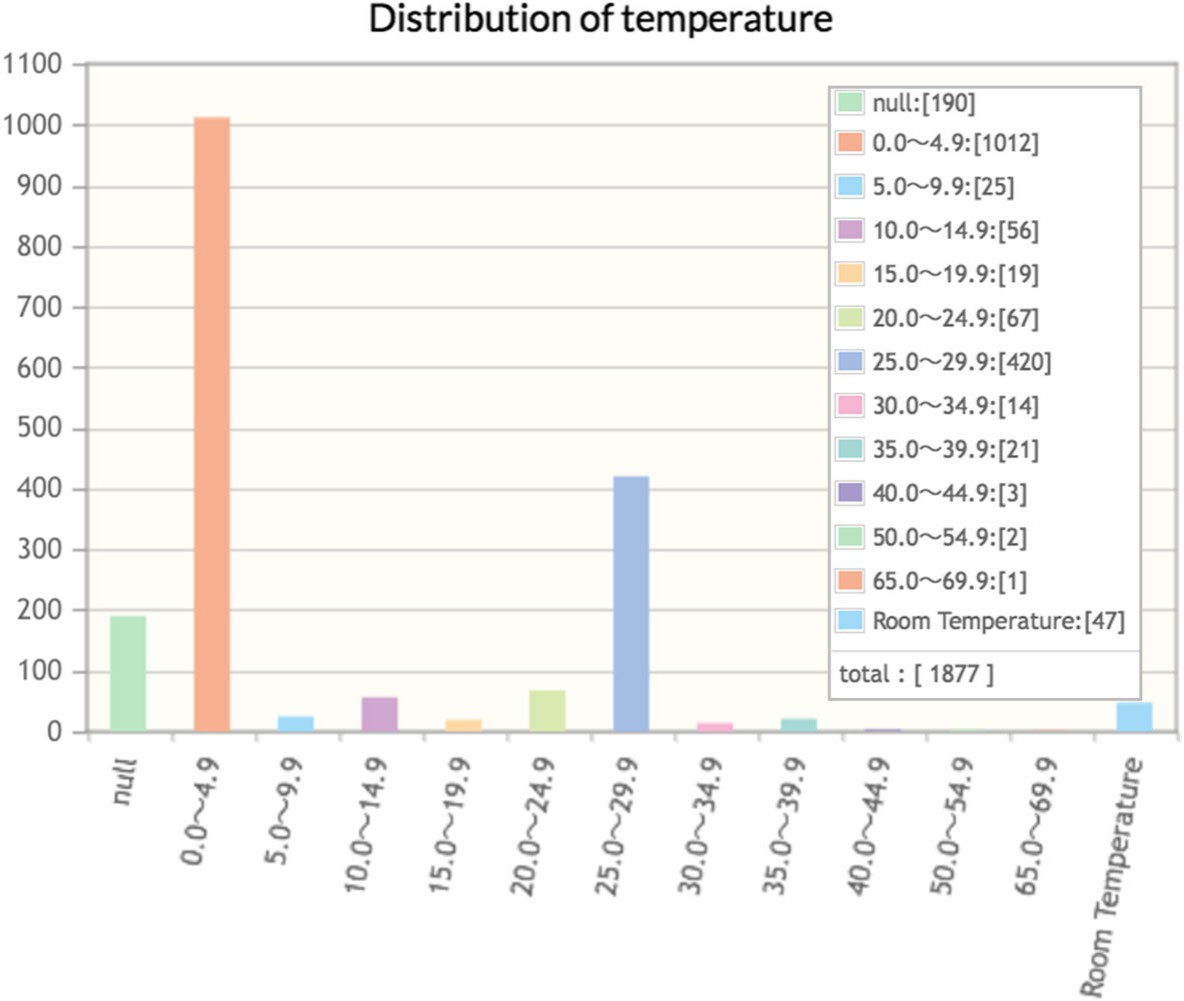
Statistics on an experimental condition, temperature
  “Statistics” on properties of proteins could also be informative for optimal experimental design, e.g. disulfide bonds, domains, isoelectric point, metal ions, size and species. Performing this analysis shows that refolding techniques are available for a protein size range comparable to that in the PDB (Fig. 6) [21]. Both graphs imply that proteins of 100–400 amino acids are frequently analyzed. It is to be noted that REFOLDdb contains a diverse cross-section of protein architectures, including extracellular domains, subunits, whole proteins and multiple proteins. It is possible in theory to transform statistics to an inference engine based on AAseqs. However, it requires stringent cleansing of AAseqs in research articles and databases that often implicitly include tags and linkers. It is also a difficult task to collect negative data that are prerequisite for the development of a reliable inference engine. Nevertheless, REFOLDdb is a good starting point for data mining in order to customize experimental conditions for a given protein in the future.

**Fig. 6:**
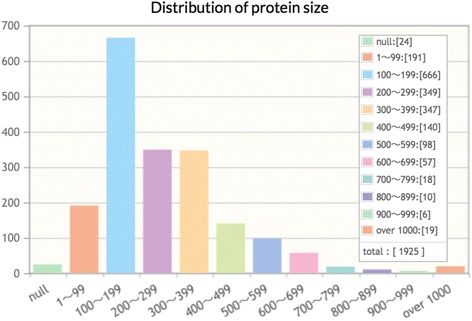
Statistics on size of proteins refolded
- The main search window (Fig. 7).

**Fig. 7:**
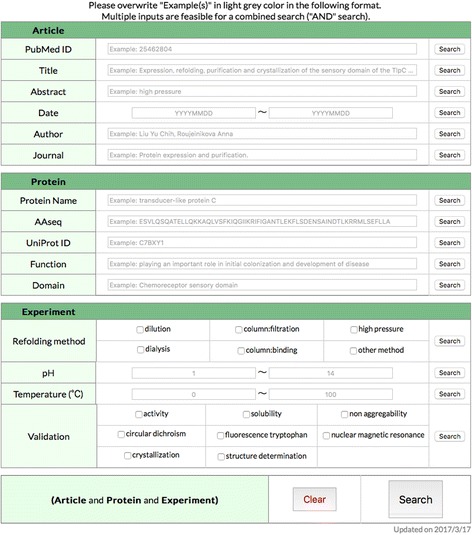
The main window for searching REFOLDdb
  The window is located just under the horizontal bar menu. A combined search of REFOLDdb can be carried out in the following two steps:
  1. Overwrite “Example(s)” in light grey color and/or check boxes as many as needed. In the case of the data items of “pH” and “Temperature”, lower limit and/or upper limit can be specified.
  2. Click a “Search” button in the line of a data item to go through the specified data item, or click the “Search” button at the bottom-right corner of the search window to perform a combined search, namely, “AND” search of multiple data items.
  Multiple hits to a query will be displayed in a table format that is composed of sortable columns of “Protein Name”, “UniProt ID”, “PubMed ID”/”Title”/”Year” of the publication, “pH”, “Temp(erature)”. “Detail” buttons in the table navigates the user to detailed information on proteins and experimental conditions.

## Conclusions

The resources, including human resources, required for running and updating REFOLDdb is kept to a minimum. A team of one annotator who is knowledgeable about structural biology and a part time system engineer will be able to keep REFOLDdb up-to-date as far as collecting data from research papers on a monthly basis. The database system based on the virtual machine is almost autonomous and also flexible enough to allow future expansion.

In the future, we will evaluate new data sources other than research articles, such as patents, that might make the database more comprehensive. In addition, we will investigate the implementation of data-mining functionality to allow the prediction of suitable refolding methods based on chemical, physical and/or genetic features of proteins that have been successfully refolded.

## Abbreviations

REFOLD database: The database on refolding technologies developed by Monash University
REFOLDdb: The database on refolding technologies developed by the authors
Tech_db: Databases on technologies
